# A matrix rank based concordance index for evaluating and detecting conditional specific co-expressed gene modules

**DOI:** 10.1186/s12864-016-2912-y

**Published:** 2016-08-22

**Authors:** Zhi Han, Jie Zhang, Guoyuan Sun, Gang Liu, Kun Huang

**Affiliations:** 1 College of Computer and Control Engineering, Nankai University, Tianjin, China; 2 College of Software, Nankai University, Tianjin, China; 3 Department of Biomedical Informatics, The Ohio State University, Columbus, OH USA; 4 The CCC Biomedical Informatics Shared Resource, The Ohio State University, Columbus, OH USA

## Abstract

**Background:**

Gene co-expression network analysis (GCNA) is widely adopted in bioinformatics and biomedical research with applications such as gene function prediction, protein-protein interaction inference, disease markers identification, and copy number variance discovery. Currently there is a lack of rigorous analysis on the mathematical condition for which the co-expressed gene module should satisfy.

**Methods:**

In this paper, we present a linear algebraic based Centralized Concordance Index (CCI) for evaluating the concordance of co-expressed gene modules from gene co-expression network analysis. The CCI can be used to evaluate the performance for co-expression network analysis algorithms as well as for detecting condition specific co-expression modules. We applied CCI in detecting lung tumor specific gene modules.

**Results and Discussion:**

Simulation showed that CCI is a robust indicator for evaluating the concordance of a group of co-expressed genes. The application to lung cancer datasets revealed interesting potential tumor specific genetic alterations including CNVs and even hints for gene-fusion. Deeper analysis required for understanding the molecular mechanisms of all such condition specific co-expression relationships.

**Conclusion:**

The CCI can be used to evaluate the performance for co-expression network analysis algorithms as well as for detecting condition specific co-expression modules. It is shown to be more robust to outliers and interfering modules than density based on Pearson correlation coefficients.

## Background

Gene co-expression network analysis (GCNA) is widely adopted in bioinformatics and biomedical research. It has many biomedical applications such as gene function prediction [1–4], protein-protein interaction inference [1, 5–7], disease markers identification [3, 8], and copy number variance discovery [9, 10]. Many GCNA algorithms have been developed to identify gene modules of strongly co-expressed genes [3, 7, 11–15]. These gene modules can be used to further infer co-regulation mechanisms such as common transcription factors as well as genetic mutations such as copy number alterations in specific chromatin regions.

Mathematically, the co-expression of the genes is often measured using correlation metrics with Pearson correlation coefficient being the most widely used one [1, 7, 11]. However, there has been a lack of rigorous analysis on the mathematical condition for which the co-expressed gene module should satisfy. As to be shown in this paper, the mathematical condition is a rather straightforward linear algebra based condition. And the condition can lead to an effective metric for characterizing the concordance of the gene expression profiles in the module. With the rigorous treatment and the effective metric, we can evaluate each module as well as the algorithm.

In addition, this metric can be used to detect condition specific co-expressed gene modules. Condition specific gene co-expression is an interesting problem and many methods have been developed to detect it [16–20]. However, most of the methods are based on first detecting differential correlation at gene-pair level such as the Fisher’s transformation and the Expected Conditional F-statistic (ECF) developed in [17]. Instead, using the new metric we developed here and the randomized test for this metric, we can detect condition specific gene co-expression holistically at the gene module level instead of just gene pairs. As demonstrated in our example on lung cancer data, this can lead to new candidates on different mechanisms for co-expression and discovery of potential new genetic variants associated with diseases such as cancers. Our preliminary results suggest that there is rich biological information contained in the co-expression relationships and the condition specificity that needs to be uncovered by deeper analysis and biological validations.

## Methods

### Rank condition of the expression profile data matrix for co-expressed genes

Given a set of *n* (≥2) genes and their expression levels over *N* (≥3) samples, the expression profiles can be expressed using a matrix

1 $$ G=\left[\begin{array}{ccc}\hfill {g}_{11}\hfill & \hfill \cdots \hfill & \hfill {g}_{1N}\hfill \\ {}\hfill \vdots \hfill & \hfill \ddots \hfill & \hfill \vdots \hfill \\ {}\hfill {g}_{n1}\hfill & \hfill \cdots \hfill & \hfill {g}_{nN}\hfill \end{array}\right]=\left[\begin{array}{c}\hfill {\boldsymbol{g}}_1\hfill \\ {}\hfill \vdots \hfill \\ {}\hfill {\boldsymbol{g}}_{\boldsymbol{n}}\hfill \end{array}\right]\in {\mathrm{\Re}}^{n\times N}, $$

with *N*-dimensional row vector ***g***_***i***_ = [*g*_*i*1_, *g*_*i*2_, …, *g*_*iN*_] be the expression profile for the *i*-th gene across the samples (*i* = 1, 2, …, *n*). If two genes *i* and *j* are perfectly co-expressed, ie, |*ρ*(***g***_***i***_, ***g***_***j***_)| = 1 where *ρ*(⋅,⋅) is the Pearson correlation coefficient between two vectors, then given the linear relationship between the two vectors, we have

2 $$ {g}_{ik}={\alpha}_{ij}\cdot {g}_{jk}+{\beta}_{ij}\ \left(k=1,2,\dots, N\right) $$

for some constants *α*_*ij*_ and *β*_*ij*_ and

3 $$ {\boldsymbol{g}}_{\boldsymbol{i}}={\alpha}_{ij}\cdot {\boldsymbol{g}}_{\boldsymbol{j}}+{\beta}_{ij}\cdot {1}_{\boldsymbol{N}}^{\boldsymbol{T}}, $$

where 1_***N***_ = [1, 1, …, 1]^*T*^ ∈ ℜ^*N*^ is *N*-dimensional. Therefore the matrix *G* can be re-written as

4 $$ G=\left[\begin{array}{c}\hfill {\boldsymbol{g}}_1\hfill \\ {}\hfill \begin{array}{c}\hfill {\boldsymbol{g}}_2\hfill \\ {}\hfill \vdots \hfill \end{array}\hfill \\ {}\hfill {\boldsymbol{g}}_{\boldsymbol{n}}\hfill \end{array}\right]=\left[\begin{array}{c}\hfill {\boldsymbol{g}}_1\hfill \\ {}\hfill \begin{array}{c}\hfill {\alpha}_{12}{\boldsymbol{g}}_1+{\beta}_{12}\cdot {1}_{\boldsymbol{N}}^{\boldsymbol{T}}\hfill \\ {}\hfill \vdots \hfill \end{array}\hfill \\ {}\hfill {\alpha}_{1n}{\boldsymbol{g}}_1+{\beta}_{1n}\cdot {1}_{\boldsymbol{N}}^{\boldsymbol{T}}\hfill \end{array}\right]=\left[\begin{array}{c}\hfill {\boldsymbol{g}}_1\hfill \\ {}\hfill \begin{array}{c}\hfill {\alpha}_{12}{\boldsymbol{g}}_1\hfill \\ {}\hfill \vdots \hfill \end{array}\hfill \\ {}\hfill {\alpha}_{1n}{\boldsymbol{g}}_1\hfill \end{array}\right]+\left[\begin{array}{c}\hfill 0\hfill \\ {}\hfill \begin{array}{c}\hfill {\beta}_{12}\cdot {1}_{\boldsymbol{N}}^{\boldsymbol{T}}\hfill \\ {}\hfill \vdots \hfill \end{array}\hfill \\ {}\hfill {\beta}_{1n}\cdot {1}_{\boldsymbol{N}}^{\boldsymbol{T}}\hfill \end{array}\right]=\left[\begin{array}{c}\hfill 1\hfill \\ {}\hfill \begin{array}{c}\hfill {\alpha}_{12}\hfill \\ {}\hfill \vdots \hfill \end{array}\hfill \\ {}\hfill {\alpha}_{1n}\hfill \end{array}\right]\cdot {\boldsymbol{g}}_1+\left[\begin{array}{c}\hfill 0\hfill \\ {}\hfill \begin{array}{c}\hfill {\beta}_{12}\hfill \\ {}\hfill \vdots \hfill \end{array}\hfill \\ {}\hfill {\beta}_{1n}\hfill \end{array}\right]\cdot {1}_{\boldsymbol{N}}^{\boldsymbol{T}}. $$

Thus the matrix *G* can be decomposed as the sum of two matrices each has rank 1. Since it has been well established in linear algebra that given two matrices *A* and *B* of the same size, *rank*(*A* + *B*) ≤ *rank*(*A*) + *rank*(*B*) [21], we have the following proposition:

#### Proposition 1

*If the absolute value of the Pearson correlation coefficient (PCC) between every pair of rows of a matrix G* (*G* ∈ ℜ^*n* × *N*^, *n* ≥ 2, *N* ≥ 3) *is 1, then rank*(*G*) ≤ 2.

Furthermore, if any of the rows of *G* is shifted or scaled (e.g., *g*_*ik*_^'^ = *λ* ⋅ *g*_*ik*_ + *ε*), the PCC value between them will still have absolute value 1 and thus the Proposition 1 still holds.

### SVD based methods for estimating the rank of *G*

Given the matrix *G*, its singular value decomposition (SVD) is *G* = *USV*^*T*^ where *U* ∈ ℜ^*n* × *n*^, *V* ∈ ℜ^*N* × *N*^ are both orthonormal matrices and *S* is a diagonal matrix with all the elements being zero except for the ones on the diagonal line which are non-negative and sorted in descending order (ie, *S*_11_ ≥ *S*_22_ ≥ … ≥ *S*_*KK*_ ≥ 0, where *K* = min(*n*, *N*)). In addition, let ‖*G*‖ be the Frobenius norm of *G* such that ${\left\Vert G\right\Vert}^2={\displaystyle \sum_{i=1}^n}{\displaystyle \sum_{j=1}^N}{g}_{ij}^2$, then it is well known that

5 $$ {\left\Vert G\right\Vert}^2={\displaystyle {\sum}_{i=1}^n{\displaystyle {\sum}_{j=1}^N{g}_{ij}^2=}}{\displaystyle {\sum}_{i=1}^K{S}_{KK}^2,}\kern0.75em K= \min \left(n,\ N\right). $$

If *G* satisfies the condition of Proposition 1, then the rank of *G* is 2 which implies *S*_33_ = *S*_44_ = … = *S*_*KK*_ = 0. Thus

6 $$ {R}_{12}=\frac{S_{11}^2+{S}_{22}^2}{{\left\Vert G\right\Vert}^2}=1. $$

In reality, given the expression profile matrix of a set of co-expressed genes, the perfect PCC value can never be reached and thus *G* is never really rank 2, but instead it can be approximated with a rank 2 matrix. Thus in theory, given an expression profile matrix *G*, we can examine if the genes (row vectors) are co-expressed by testing if the ratio *R*_12_ defined in (6) is close to 1. We refer *R*_12_ as the *concordance index*.

### Data transformation and centralized concordance index

While the concordance index *R*_12_ can be used as a potential indicator for the concordance of the rows of *G* and thus for evaluating co-expressed modules, it is difficult to set a hard threshold for it. This is even more challenging for real data due to noise, batch effects, and background signals that may skew the PCC values. In addition, since SVD is based on the *L*^2^ -norm, it can be biased by any specifically large outlier or just a few genes with high expression levels. Thus the data needs to be transformed before processing. The transformation of the data we proposed involves two steps: centralization and standardization. First, each row of *G* needs to be centralized by subtracting its average such that

7 $$ \overline{G}=\left[\begin{array}{ccc}\hfill {g}_{11}-\overline{g_1}\hfill & \hfill \cdots \hfill & \hfill {g}_{1N}-\overline{g_1}\hfill \\ {}\hfill \vdots \hfill & \hfill \ddots \hfill & \hfill \vdots \hfill \\ {}\hfill {g}_{n1}-\overline{g_n}\hfill & \hfill \cdots \hfill & \hfill {g}_{nN}-\overline{g_n}\hfill \end{array}\right]=\left[\begin{array}{c}\hfill {\boldsymbol{g}}_1^{\boldsymbol{c}}\hfill \\ {}\hfill \vdots \hfill \\ {}\hfill {\boldsymbol{g}}_{\boldsymbol{n}}^{\boldsymbol{c}}\hfill \end{array}\right],\  where\ \overline{g_i}=\frac{{\displaystyle {\sum}_{k=1}^N}{g}_{ik}}{N}\ for\ i=1,2,\dots, n. $$

Next, each row of $\overline{G}$ is standardized to have norm 1, ie

8 $$ \widehat{G}=\left[\begin{array}{c}\hfill \raisebox{1ex}{${\boldsymbol{g}}_1^{\boldsymbol{c}}$}\!\left/ \!\raisebox{-1ex}{$\left\Vert {\boldsymbol{g}}_1^{\boldsymbol{c}}\right\Vert $}\right.\hfill \\ {}\hfill \vdots \hfill \\ {}\hfill \raisebox{1ex}{${\boldsymbol{g}}_{\boldsymbol{n}}^{\boldsymbol{c}}$}\!\left/ \!\raisebox{-1ex}{$\left\Vert {\boldsymbol{g}}_{\boldsymbol{n}}^{\boldsymbol{c}}\right\Vert $}\right.\hfill \end{array}\right]=\left[\begin{array}{c}\hfill {\widehat{\boldsymbol{g}}}_{\mathbf{1}}\hfill \\ {}\hfill \vdots \hfill \\ {}\hfill {\widehat{\boldsymbol{g}}}_{\boldsymbol{n}}\hfill \end{array}\right],\  where\ {\widehat{\boldsymbol{g}}}_{\boldsymbol{k}}=\raisebox{1ex}{${\boldsymbol{g}}_{\boldsymbol{k}}^{\boldsymbol{c}}$}\!\left/ \!\raisebox{-1ex}{$\left\Vert {\boldsymbol{g}}_{\boldsymbol{k}}^{\boldsymbol{c}}\right\Vert $}\right.,\ k=1,2,\dots, n. $$

The centralization step aims to mitigate the background signal while the standardization step avoids bias towards any particularly highly expressed genes. Interestingly, since the Pearson correlation coefficient between ***g***_***i***_ and ***g***_***j***_ is defined as

9 $$ \rho \left({g}_i,\ {g}_j\right)=\frac{{\displaystyle {\sum}_{k=1}^N}\left({g}_{ik}-{\overline{g}}_i\right)\cdot \left({g}_{jk}-{\overline{g}}_j\right)}{\sqrt{{\displaystyle {\sum}_{k=1}^N}{\left({g}_{ik}-{\overline{g}}_i\right)}^2}\cdot \sqrt{{\displaystyle {\sum}_{k=1}^N}{\left({g}_{jk}-{\overline{g}}_i\right)}^2}}=\frac{{\boldsymbol{g}}_{\boldsymbol{i}}^{\boldsymbol{c}}\cdot {\left({\boldsymbol{g}}_{\boldsymbol{j}}^{\boldsymbol{c}}\right)}^{\boldsymbol{T}}}{\left\Vert {\boldsymbol{g}}_{\boldsymbol{i}}^{\boldsymbol{c}}\right\Vert \cdot \left\Vert {\boldsymbol{g}}_{\boldsymbol{j}}^{\boldsymbol{c}}\right\Vert }={\widehat{\boldsymbol{g}}}_{\boldsymbol{i}}\cdot {\left({\widehat{\boldsymbol{g}}}_{\boldsymbol{j}}\right)}^{\boldsymbol{T}} $$

and ‖***ĝ***_***k***_‖ = 1 (*k* = 1, 2, …, *n*), therefore |*ρ*(*g*_*i*_, *g*_*j*_)| = 1 implies ***ĝ***_***k***_ = ***ĝ***_1_ or ***ĝ***_***k***_ = − ***ĝ***_1_. In other words,

$$ \widehat{G}=\left[\begin{array}{c}\hfill {\alpha}_1\hfill \\ {}\hfill \vdots \hfill \\ {}\hfill {\alpha}_n\hfill \end{array}\right]{\hat{\boldsymbol{g}}}_1,\  where\ {\alpha}_i\in \left\{+1, - 1\right\}\ for\ i=1,2,\dots, n. $$

Therefore we have the following proposition:

#### Proposition 2

*If the absolute value of the Pearson correlation coefficient (PCC) between every pair of rows of a matrix G* (*G* ∈ ℜ^*n* × *N*^, *n* ≥ 2, *N* ≥ 3) *is 1 and Ĝ is the centralized matrix of G with each row standardized as in (*8*), then rank*(*Ĝ*) = 1. *In addition, the inner product between every pair of rows equals the Pearson correlation coefficient of the two rows.*

Thus the singular value decomposition (SVD) for *Ĝ* is $\widehat{G}=\widehat{U}\widehat{S}{\widehat{V}}^T$ where $\widehat{U}\in {\mathrm{\Re}}^{n\times n},\ \widehat{V}\in {\mathrm{\Re}}^{N\times N}$ are both orthonormal matrices and *Ŝ* is a diagonal matrix with all the elements being zero except for the ones on the diagonal line which are non-negative and sorted in descending order (ie, *Ŝ*_11_ ≥ *Ŝ*_22_ ≥ … ≥ *Ŝ*_*KK*_ ≥ 0, where *K* = min(*n*, *N*)). In fact, given that *Ĝ* is rank 1 and *Ĝ* = *n*, we have

11 $$ {\widehat{S}}_{11}^2=n\ \mathrm{and}\ {\widehat{S}}_{22}=\dots ={\widehat{S}}_{KK}=0. $$

We therefore define a *centralized concordance index* (CCI) as

$$ CCI=\frac{{\widehat{S}}_{11}^2}{n}. $$

### Estimate the significance of the CCI

We examine two approaches for determining if the observed *CCI* is significantly large to reflect co-expression relationship among the ***n*** genes over the entire whole genome dataset. First, we randomly permute the entries of every row of *Ĝ* and calculate *CCI*_*p*_. This process is repeated M times (usually we choose M = 1000). Then we set

$$ {p}_{permute}=\#\left(CC{I}_p\ge CCI\right)/M. $$

Conceptually this gives a measurement on how significant is the observed concordance index in the background of the same data distribution.

**Fig. 1:**
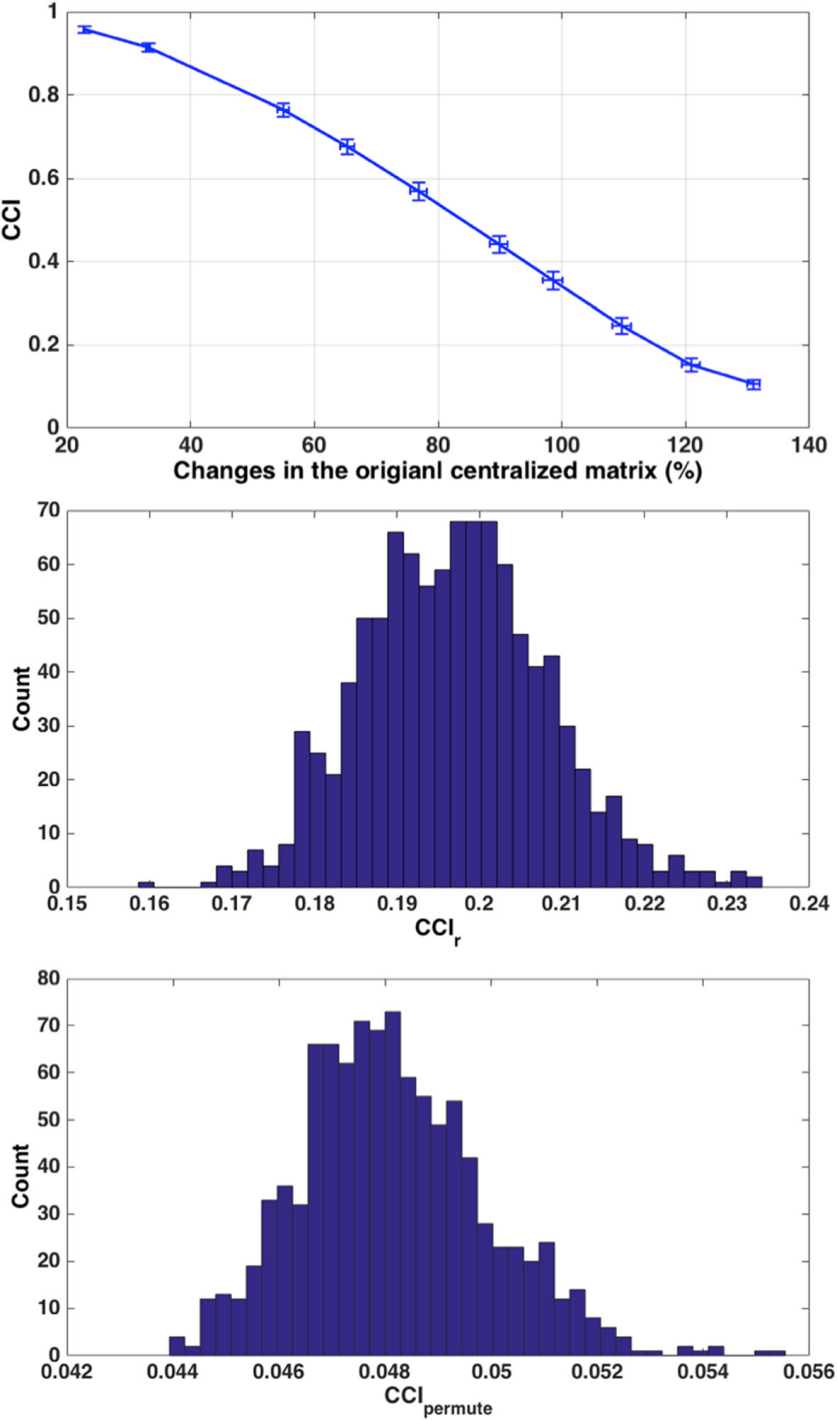
Simulation on the distribution of CCI and its relationship with noise in the data. *Top*: Relationship between CCI and noise level. The x-axis reflects the effects of the noise on the centralized matrix. *Middle*: The distribution of CCI calculated from 1000 randomly selected gene lists (with 220 genes) in the 41 lung cancer tumor samples (using GSE18842). *Bottom*: The distribution of CCI calculated from 1000 random permutation of the data from the correlated gene module

**Fig. 2:**
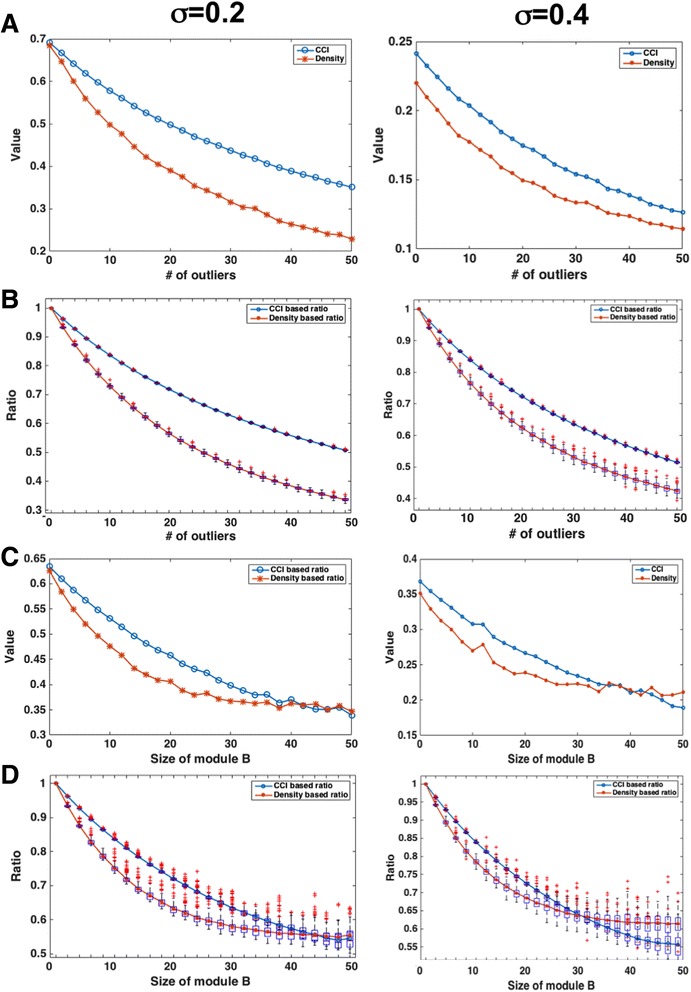
Comparison between CCI and density metrics. **a** The CCI versus density metrics with the increases of number of outliers under two different noise levels. **b** The boxplots for the two metrics with different number of outliers and noise levels. The values are normalized to the values with zero outlier. **c** The CCI versus density metrics with the increasing size of the interfering module. **d** The boxplots for the two metrics with different number of outliers and noise levels with the values normalized to the values without interfering module

Next, we randomly choose ***n*** genes from the whole genome gene expression data and calculate the *CCI*_*r*_. This process is repeated M times. We then calculate the z-score *Z*_*CCI*_ for the *CCI* based on the random sampling such that ${Z}_{CCI}=\frac{CCI- mean\left(CC{I}_r\right)}{std\left(CC{I}_r\right)}$. The significance is then estimated from the z-score. This gives a measurement on how significant is the observed CCI for the tested gene module in the entire genome. We choose z-score instead of the percentile of the CCI due to three reasons: 1) simulation and tests on real data shows that *CCI*_*r*_ follow a bell-shaped distribution which can be reasonably approximated by a Gaussian distribution as shown in Figs. 1 and 2) even with 1000 times sampling, it is still relatively small comparing to all the possible combinations, thus sometimes although CCI is larger than all *CCI*_*r*_, it is not reasonable to assume that the *p*-value (significance) is extremely small, instead z-score gives a reasonable estimate on the deviation of the observed CCI from the random samples; and 3) last but not the least, one of our goals is to use the metric to compare results from different conditions, z-scores are standardized with the same distribution and thus allows us to compare with different conditions.

### Simulation

To evaluate the performance of the concordance index, we generate a matrix of 50 × 100 with absolute value of PCC between every pair of rows being 1. The base vector is generated as a 100-dimensional row vector using uniform distribution from 0 to 1. The scaling factors (*α*) and shifts (*β*) are also generated using uniform distribution from 0 to 1. The matrix *G* is calculated using Eq.(4). Then Gaussian noises with zero mean at different levels (*σ* = 0.01, 0.02, 0.05, 0.07, 0.1, 0.15, 0.2, 0.3, 0.5, 1) are added to the matrix and corresponding concordance indices are calculated. This process is repeated 1000 times for each noise level. In addition, for each test the centralized matrix *Ĝ*_*r*_ was compared with the original *Ĝ* using ratio ${R}_F=\frac{\left\Vert {\widehat{G}}_r-{\widehat{G}}_F\right\Vert }{\left\Vert \widehat{G}\right\Vert F}$, where ‖ ⋅ ‖_*F*_ is the Frobenius norm of the matrix.

### Comparison with density metric

Since a focus of mining co-expression network is to identify densely connected gene modules, the metric density defined for network mining is often used. For a module with ***n*** in weighted network, its density is defined as

$$ d=\frac{{\displaystyle {\sum}_{i=1}^{n-1}}{\displaystyle {\sum}_{j=i+1}^n}{w}_{ij}}{n\left(n-1\right)/2}. $$

For co-expression networks, the weight *w*_*ij*_ is often defined as the correlation coefficient |*ρ*(*g*_*i*_, *g*_*j*_)| or its transformation. In order to examine the relationship between CCI and density, we compare CCI with the density defined using |*ρ*(*g*_*i*_, *g*_*j*_)| as weights. Specifically, we consider two scenarios. The first is to test the responses of the metrics to outliers. We first generate the simulated matrix *G* as described above. Then outlier (independently generated vectors) will be added. We calculate both metrics under different number of outliers and different noise levels for *G*. The second scenario is to consider the possibility that two modules sometimes can be erroneously linked together. To test this, we generate two gene modules and test the responses of the two metrics with respect to different sizes and noise levels of the modules. Each test is repeated 100 times.

### Datasets

We test the concordance index and its significance using a large gene expression dataset. The dataset was downloaded from NCBI Gene Expression Omnibus (GEO). The dataset is GSE18842 containing gene expression microarray data from 46 non-small cell lung cancer (adenocarcinoma) tumor samples and 45 non-cancer control tissue samples [22]. The GSE18842 dataset was generated using Affymetrix HU133 2.0Plus GeneChip. The normalization of the dataset was verified by inspecting the boxplot and data distributions. We also tested some of the findings using TCGA non-small cell lung cancer adenocarcinoma [23] and squamous cell tumor data [24] from cBioPortal (http://www.cbioportal.org/).

### Weighted co-expression network analysis

While the R package WGCNA developed by the Horvath’s group is a widely adopted co-expression gene module discovery tool, it has some limitation as it is based on hierarchical clustering algorithm that does not allow overlap between modules and does not control the density of the detected modules [11]. In this paper, we apply a recently developed algorithm called Normalized lmQCM [15]. This algorithm takes a network mining approach allowing overlaps between modules and also is guaranteed to have a lower bound on the density of the detected modules.

### Using CCI to detect condition specific modules

The concordance index and its significance evaluation provide us a means to evaluate if a co-expressed gene module (CGM) in one condition is also strongly co-expressed in another condition. We first apply the Normalized lmQCM to each conditions (normal and disease) in both datasets using selected parameters (to be discussed in the Results section). For each gene module, we then calculated two CCIs, one using the data from the condition it was generated and one using the data from the opposite condition. For instance, if the module was generated from the Parkinson’s disease patients, CCIs for the same gene module is calculated for both Parkinson’s disease samples and the control sample. Then the *p*_*permute*_ and *Z*_*CCI*_ are calculated for both conditions too. Gene modules that are significant (*Z*_*CCI*_ ≤ *τ*) in one condition but not significant (*Z*_*CCI*_ > *τ*) in the other condition are reported for further analysis. The threshold *τ* is determined based on the significance requirement. For instance, *τ* is often chosen such that the one-tail *p*-value for the *Z*_*CCI*_ is less than 0.05 for single gene module or 0.05/*m* if *m* gene modules are being tested (for multiple test compensation).

### Enrichment analysis for gene modules

For the reported modules, we carry out enrichment analysis using TOPPGene (https://toppgene.cchmc.org/enrichment.jsp). We specifically pay attention to significantly enriched Gene Ontology (GO) terms, cytobands, transcription factor binding sites, and human/mouse phenotypes.

## Results

### Simulation on the relationship between CCI

As described in the Methods section, we generated the matrix with correlated rows. The matrix *G* was then calculated using Eq. (4). Then Gaussian noises with zero mean at different levels (*σ* = 0.01, 0.02, 0.05, 0.07, 0.1, 0.15, 0.2, 0.3, 0.5, 1) were added to the matrix and corresponding concordance indices were calculated. This process is repeated 1000 times for each noise level. In addition, for each test the centralized matrix *Ĝ*_*r*_ was compared with the original *Ĝ* using ratio ${R}_F=\frac{\left\Vert {\widehat{G}}_r-{\widehat{G}}_F\right\Vert }{\left\Vert \widehat{G}\right\Vert F}$, where ‖ ⋅ ‖_*F*_ is the Frobenius norm of the matrix. The relationship between the CCI and the difference between the noisy matrix with the original matrix is plotted in Fig. 1 Top.

We then tested the distribution of the CCI in random gene lists using real data. As shown in Fig. 1 Middle, 1000 randomly selected gene lists with 220 genes (based on a real module with CCI 0.4957 generated from the co-expression analysis) in 41 lung cancer tumor samples from GSE18842 were generated and the distribution of the CCI follow a bell shaped curve with a mean of 0.1974 and standard deviation of 0.0117. Thus *z*_*CCI*_ is 25.49. In addition we carried out 1000 times of random permutation of the data from the co-expressed gene module with 220 genes and the distribution is shown in Fig. 1 Bottom. The permutation results follow a tight distribution with mean of 0.0482 and standard deviation of 0.00176. While this clearly shows that the observed CCI (0.4957) is not associated with the data distribution, the fact that these permutation results are much lower than randomly picked gene sets from the original dataset (as shown in Fig. 1 Middle) suggests the permutation test practically cannot provide new information regarding the significance of the modules. Therefore in the rest of the paper we focus on the z-score based approach from random gene list to evaluate the modules. Similar distributions were observed in multiple datasets with different number of genes or sample sizes (data not shown).

### Comparison with density metric

As described in the Methods section, we first consider the scenario when the different numbers of “outlier vectors” were added to the correlated matrix *G* with 50 rows and 100 columns. Figure 2a shows two instances of the simulation for different choices of the noise level. In both cases, the metrics (CCI and density) decrease as the number of outliers increases. However, it can be seen that the curve for the CCI is smoother than the curve for the density, suggesting that CCI is more robust in response to outliers. This is further confirmed in Fig. 2b when the ranges of the values for both metrics over the 100 times simulation are plotted. In Fig. 2b, the values of the metrics are normalized according to the value of zero outlier. It is clear that the ranges for CCI are always tight when the density values span a wide range. Similar results are observed for the two-module scenario as shown in Fig. 2c and d. In addition, it is clear that with the increase of size of the interfering module, the density is no long sensitive when the size of the interfering module is more than half of the original module while the CCI consistently decreases.

### Identify tissue specific co-expressed modules in lung tumor

We first carried out weighted gene co-expression network mining using the normalized lmQCM algorithm on the lung tumor data (41 samples) using parameter *γ* = 0.4. *γ* is a major parameter for the normalized lmQCM algorithm. The larger its value, the higher is the density of the identified gene modules. Our previous study suggested *γ* = 0.4 is a reasonable values for such dataset.

**Fig. 3:**
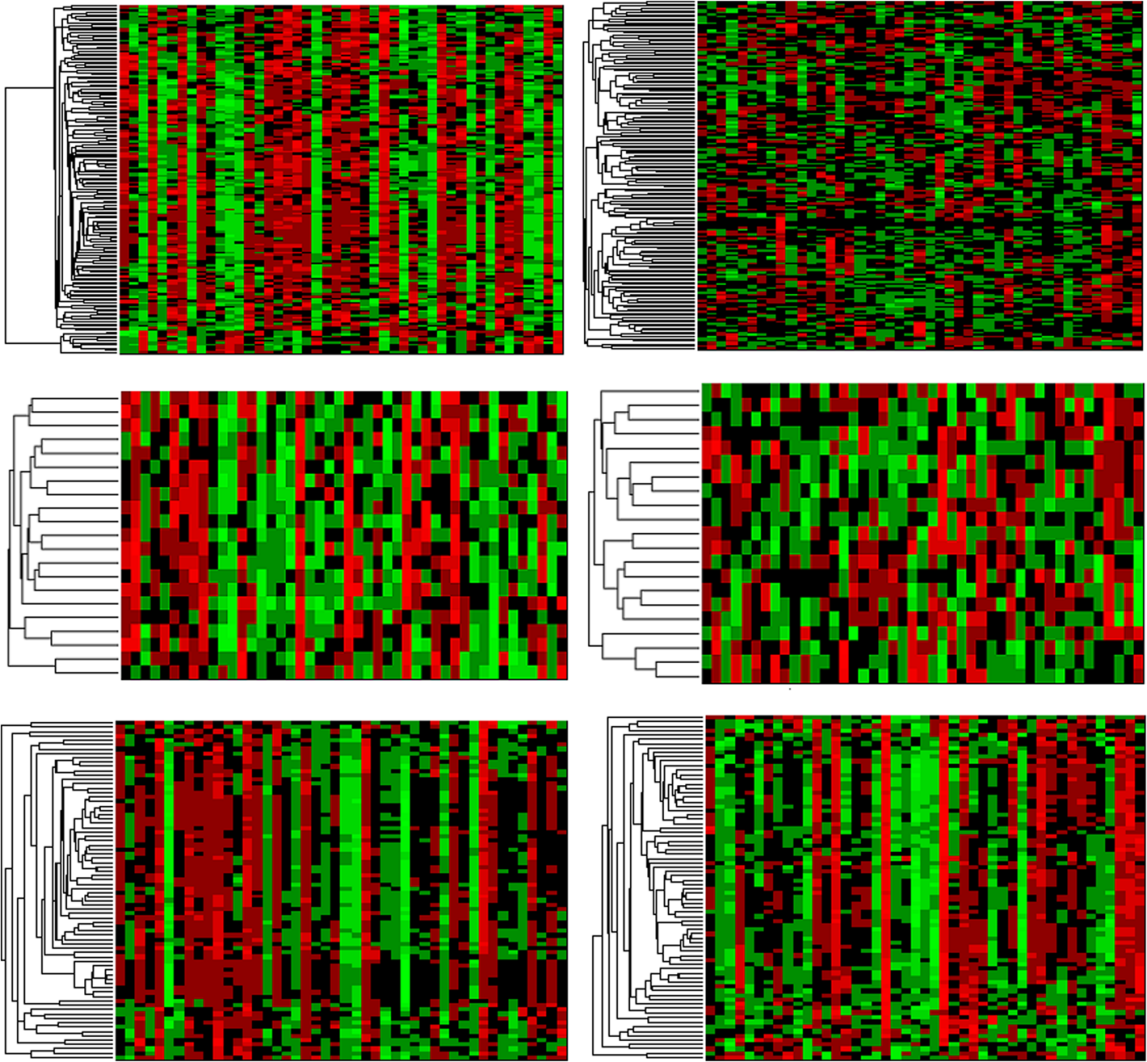
Examples of the gene modules in tumor samples (*left column*) and control samples (*right column*). The top two modules show significant difference in co-expression between control and tumor samples with high CCIs and z-scores in tumor and low CCIs as well as low z-scores in control samples. The bottom module has high CCIs and z-scores in both tumor and control samples

The algorithm yielded 168 gene modules with at least five genes (ranging from five to 891). We then calculated the CCI and z-score based on 1000 randomly selected gene lists of the same size for every module. Then we calculated the CCI and z-score for the same set of gene lists in the control samples. We selected the threshold for z-score to be 3.433 such that the one-tail *p*-value is less than 0.05/168 = 0.000298. Among the 168 gene modules, all the z-scores derived from the tumor samples are higher than the threshold while 15 of the gene modules have z-scores lower than the threshold in control samples. Figure 3 shows the heatmaps of three examples of the gene modules. Two (Figure 3 Top and Middle) have high z-scores in tumor samples but lower than threshold z-scores in control samples. This is also reflected in the heatmap. In the tumor samples (Fig. 3 Left), the expression levels of the samples show clear consistent patterns across the samples while there is no clear pattern in the control samples. The last module in Fig. 3 (bottom) has high CCIs and z-scores in both tumor and control samples and it is clear the expression levels of the genes show consistent patterns in both cohorts.

These 15 gene modules are further analyzed for enriched biological processes, cytobands and transcription factor binding sites. Table 1 summarizes the findings from these 15 gene modules.

**Table 1:** Enrichment analysis of the 15 gene modules that are specific to tumor samples in lung cancer

Module	Size	GO BP term (*p*-value)	Cytoband (*p*-value)	TF (*p*-value)
4	162	Epidermis development (*p* = 8.762E-23);	1q21-q22 (*p* = 3.354E-06);	AP1 (*p* = 2.177E-04, 22 genes);
			2p24.3 (*p* = 5.021E-06)	AREB6 (*p* = 9.900E-04, 8 genes)
5	159	neuron differentiation (*p* = 1.516E-07);	6q14.2 (*p* = 1.796E-04);	PAX4 (*p* = 1.046E-05, 9 genes);
		generation of neurons (*p* = 2.105E-07)	5q33 (*p* = 2.686E-04)	MSX1 (*p* = 2.088E-04, 7 genes)
9	98	Neurogenesis (*p* = 1.490E-06);		MSX1 (*p* = 2.875E-06, 7 genes);
		central nervous system development (*p* = 2.543E-06)		RNGTGGGC UNKNOWN (*p* = 3.383E-05, 11 genes)
17	62	meiotic nuclear division (*p* = 1.035E-04);	7p15.3-p15.1 (*p* = 1.676E-03);	
		meiotic cell cycle (*p* = 1.349E-04)	12q22-q24.1 (*p* = 1.676E-03)	
19	55	cellular glucuronidation (*p* = 2.634E-05);	4q13 (*p* = 2.202E-04);	
		uronic acid metabolic process (*p* = 3.005E-05)	4q31.3-q32 (*p* = 1.474E-03)	
25	48	glutamate decarboxylation to succinate (*p* = 4.577E-06);	4q21.22 (*p* = 1.646E-06);	
		glutamate catabolic process (*p* = 9.526E-05)	8p11.22 (*p* = 1.485E-04)	
38	36	calcium ion export (*p* = 2.626E-05)	7q21.3 (*p* = 8.602E-06);	
			9p21.3 (*p* = 2.647E-04)	
44	33	vasodilation of artery involved in baroreceptor response to increased systemic arterial blood pressure (*p* = 2.381E-06);	7p12.2 (*p* = 1.729E-05);	RORA1 (*p* = 6.429E-03, 3 genes);
		baroreceptor response to increased systemic arterial blood pressure (*p* = 1.424E-05)	11p15.2-p15.1 (*p* = 9.241E-04)	ERR1 (*p* = 7.357E-03, 3 genes)
50	30	fatty acid derivative metabolic process (*p* = 1.751E-07);	4q28-q32 (*p* = 1.673E-03)	WGTTNNNNNAAA UNKNOWN (*p* = 2.278E-03, 4 genes);
		icosanoid metabolic process (*p* = 1.751E-07)		FOXO4 (*p* = 1.168E-02, 6 genes)
66	21		4p16.3 (*p* = 2.197E-9, 5 genes); 13 genes on 4p13-16	E2F1 (*p* = 9.854E-4, 3 genes)
67	21		9q21.33 (*p* = 5.973E-05);	RACTNNRTTTNC UNKNOWN (*p* = 3.031E-05, 3 genes)
			9q22.32 (*p* = 1.652E-04); 18 genes on 9q21-34	
70	20		1q22-q23.2 (*p* = 5.196E-04)	
			8q22-q23 (*p* = 1.038E-03)	
81	18		21q22.3 (*p* = 2.655E-05)	
84	18		4q31.23 (*p* = 6.329E-06); 4q31 (*p* = 1.242E-05); 12 genes on 4q23-31	CREB (*p* = 1.568E-04, 3 genes)
116	13		Xp11.23 (*p* = 6.422E-04)	MEIS1 (*p* = 1.089E-03, 3 genes)

## Discussion

One important issue is the biological mechanism leading to the differences in co-expression structures between the tumor and the control samples. As shown in Table 1, it is clear that there are multiple possible mechanisms. From the functional point of view, the first gene module (Module 4) is highly enriched in epidermis development function. This is consistent with the fact that lung cancer is an epithelial cancer. However the molecular mechanism for such difference is still not clear. While it is often expected that such difference may be due to difference in transcription factors (TFs) which co-regulate the co-expressed genes, our analysis (data not shown) on the enriched TFs shown in Table 1 did not reveal any statistically significant increase in level of the TFs in tumor samples.

**Fig. 4:**
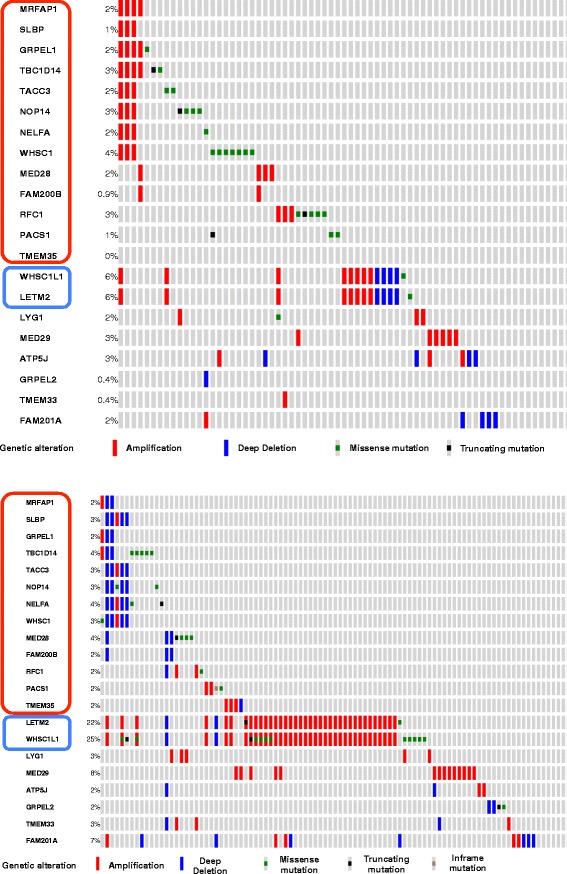
The OncoPrint plots for different types of mutations on the genes in module 66 in the lung adenocarcinoma patients. *Top*: OncoPrint for genes in Module 66 (with 21 genes) in the lung adenocarcinoma study in TCGA generated generated by cBioPortal. *Bottom*: Oncoprint for the same gene module in lung squamous cell tumors in TCGA. The genes circled in red are all on cytobands 4p13-16 and the ones circled in blue are on cytoband 8p11.23

Another possible mechanism of co-expression is that the genes may lie on the same cytoband with copy number variations (CNV) among the tumor samples. We have indeed observed a few such gene modules including modules 66 (13 genes on 4p13-13), 67 (18 genes on 9q21-34), and 84 (12 genes on 4q23-31). The difference between the tumor and control samples implies that the potential CNV may be specific to the tumor. We tested the module 66 on TCGA lung cancer data using cBioPortal. In addition to the lung adenocarcinoma data with 230 patients, we also tested on the lung squamous cell data with 178 patients. Figure 4 shows the OncoPrint plots for the distribution of different types of mutations on the genes in module 66 in the patients.

**Fig. 5:**
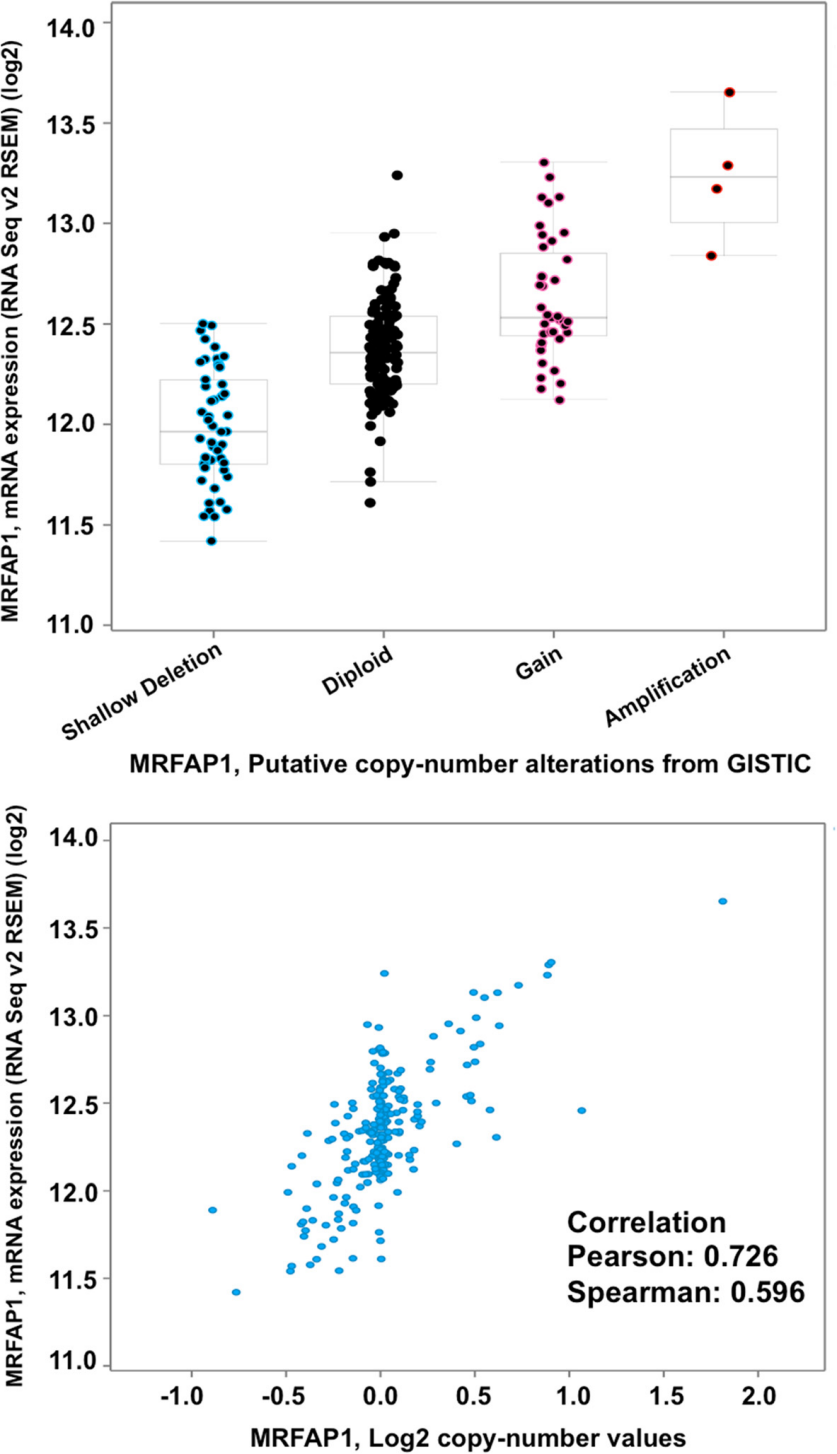
Correlations between the copy number measurements and the gene expression levels (measured using RNA-seq) of gene MRFAP1. *Top*: The box plot for the expression levels of gene MRFAP1 with respect to inferred copy number variation. *Bottom*: The correlation between the expression levels of MRFAP1 with the measurement for copy number values is 0.726 (PCC)

As shown in Fig. 4, the majority of the genes identified in Module 66 on cytobands 4p13-16 showed consistent CNV in lung cancer patients of both types. However, they are all amplifications in adenocarcinoma while mostly deletion in squamous cell tumors. To verify the relationship between the CNV and gene expression levels, we examined the correlations between the copy number measurements and the gene expression levels (measured using RNA-seq) of these genes and they all show positive correlations with an example (for the MRFAP1 genes) showing Fig. 5.

**Fig. 6:**
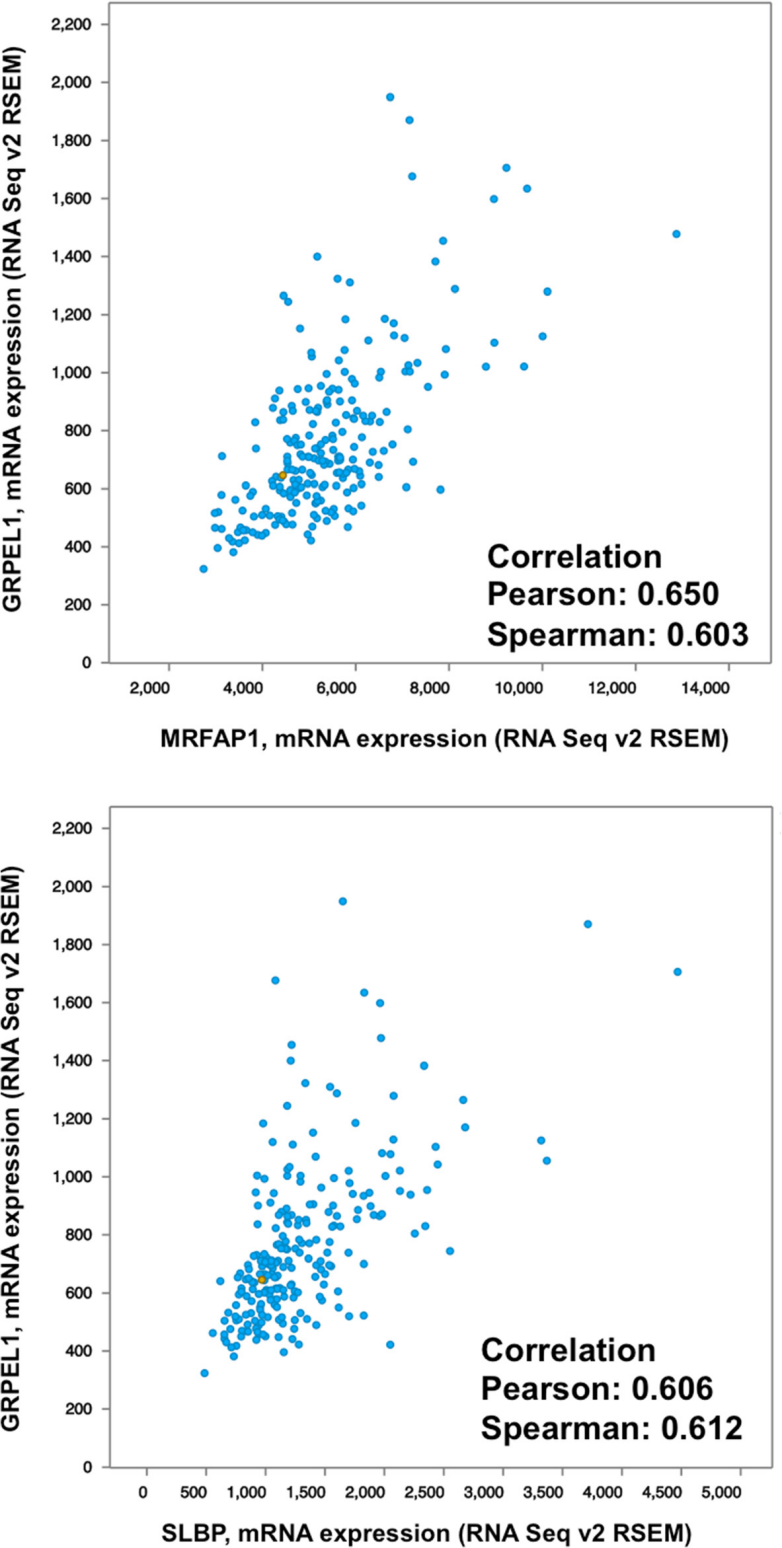
Examples of co-expressed genes on the same cytobands from the same gene module. *Top*: The correlation between expression levels of MRFAP1 and GRPL1 is 0.650 (PCC). *Bottom*: The correlation between the expression levels of SLBP and GRPL1 is 0.606 (PCC)

In addition, the genes which are on close cytobands with similar CNV distribution in patients show strong co-expression as shown in Fig. 6 while the ones not on the same cytobands do not (data not show, the correlation ranges from 0.3 to less than 0). These observations suggest that the expression levels and co-expression of the genes on these cytobands are strongly associated with the CNV status of these bands. However, we also observed difference in correlation in the original dataset GSE18842 and the testing TCGA dataset. This could be partially due to difference in sample selections and measurement methods (GSE18842 data were generated using Affymetrix genechips while TCGA expression data were generated using RNA-seq).

An additional interesting observation is that in both lung adenocarcinoma and squamous cell tumor samples, two genes from cytoband 8p11.23 show consistent copy number aberrations in the patients. While the mechanism for their co-expression with the ones on cytoband 4p1 is not clear, literature review shows that the gene TACC3 in Module 66 on cytoband 4p16 is known to have a gene fusion with FGFR1 gene in 3 % of glioblastoma multiforme patients [25]. FGFR1 gene happens to locate on 8p11.23-22. It is of great interest for future research to investigate if the relationship between the 4p16 and 8p11.23 is partially due to a gene fusion event.

## Conclusion

In summary, we have developed a linear algebraic based index CCI for evaluating the concordance of co-expressed gene modules from gene co-expression network analysis. The CCI can be used to evaluate the performance for co-expression network analysis algorithms as well as for detecting condition specific co-expression modules. It is shown to be more robust to outliers and interfering modules than density based on Pearson correlation coefficients. We applied CCI in detecting lung tumor specific gene modules. The application revealed interesting potential tumor specific genetic alterations including CNVs and even hints for gene-fusion. Deeper analysis required for understanding the molecular mechanisms of all such condition specific co-expression relationships.
